# Animal Venoms Targeting Cellular Mechanisms: Advances and Implications for Drug Discovery and Disease Therapy

**DOI:** 10.3390/toxins18070286

**Published:** 2026-06-30

**Authors:** Lucienne Mathai, Bipin G. Nair, Aswathy Alangode

**Affiliations:** School of Biotechnology, Amrita Vishwa Vidyapeetham, Amritapuri, Kollam 690525, India

**Keywords:** zootoxins, drug discovery, human diseases, artificial intelligence, nanotechnology

## Abstract

Animal venoms are complex biochemical systems composed of proteins, peptides, and small molecules with bioactivity. Traditionally regarded as toxic agents, venom components are increasingly recognized as valuable molecular libraries that can modulate cellular processes in human disease pathophysiology. This review highlights the translational potential of venom-derived molecules, with emphasis on clinically approved venom-derived drugs such as captopril, while also discussing their therapeutic potential for diseases such as cancer, autoimmunity, cardiovascular diseases, chronic pain, neuropsychiatric disorders, and infectious diseases. Special emphasis is given to the mechanisms by which these toxins modulate ion channels, enzymes, and receptor systems. Recent advances in venomics, harnessing proteomics, transcriptomics, and high-throughput screening, are also discussed, which can accelerate the clinical translation of venom-derived therapeutics. Key challenges related to assessing the immunogenicity of the venom-derived compounds, bioavailability, and safety have also been addressed alongside emerging strategies to overcome them. Collectively, these advances can provide a logical framework for developing novel therapeutics, bridging the gap between toxinology and drug discovery.

## 1. Introduction

Animal venoms are complex, specialized biochemical secretions composed of proteins, glycoproteins, enzymes, peptides, and small molecules, collectively known as toxins, that evolved for different functions, such as feeding and digestion, predation, and self-defense [1]. Venom systems are the result of adaptive evolution, which has driven morphological, biochemical, behavioral, and physiological changes in response to ecological and environmental cues [2]. Morphological traits like fangs, claws, or structures aid in feeding or self-defense; biochemical ones involve producing a toxic secretion, which has relevance in attacking or hunting to immobilize prey or avoid predators [3]. Venom consists of bioactive components that can act individually or synergistically to modulate a range of physiological processes [1,3]. When venom toxins are isolated, purified, and characterized, they have been shown to modulate several physiological and cellular pathways, including cell signaling, neuronal and cardiovascular processes, inflammatory and immune responses, blood coagulation, apoptosis, necrosis, and other mechanisms associated with human disease, making them valuable tools for drug discovery and therapeutic development [4]. Studies on animal venom composition have revealed remarkable diversity in composition and function, providing insights into the prey-predator dynamics and human disease biology [5]. Animal venoms are now recognized as not just harmful toxins but as valuable molecular toolkits with their ability to bind enzymes, ion channels, and cell membrane components such as lipids with high affinity and specificity, thereby modulating cellular and physiological processes [6,7]. This shows that they are valuable molecular libraries, from which a diverse range of drug candidates can be identified for therapeutic applications [8,9,10].

The scope of this review is to elucidate an integrated perspective of the therapeutic potential of animal venoms, highlighting that venom-derived molecules have transitioned from being identified as toxic agents to valuable pharmacological tools [11]. The review comprehensively explores the currently approved drugs from venom sources, their mechanisms of action, and the potential emerging candidates that can be used in clinical investigations against an array of conditions, such as cardiovascular diseases, cancer [7], autoimmune disorders, infectious diseases, and chronic pain [12,13,14].

### Literature Search Strategy

A comprehensive literature search was carried out using PubMed, ScienceDirect, SpringerLink, and Google Scholar. Key words included “animal venom”, “snake venom”, “spider venom”, “scorpion venom,” “venom derived drugs”, “toxins”, “enzyme toxins”, “venomics”, “toxinology”, “phospholipases A2”, “L-amino acid oxidases”, “therapeutic uses,” “peptides”, “translating venom components,” “drug discovery”, “ion channels,” “receptors,” “nociception”, “anticancer,” “antimicrobial”, “autoimmunity,” “analgesics”. Relevant combinations of these keywords were used in the literature search to identify key points on the topic discussed. Only peer-reviewed articles published in English were considered from the period of 2000–2025. However, those publications that described landmark venom-derived historical discoveries that include captopril, eptifibatide, exenatide, and ziconotide were considered from papers irrespective of the publication year. Original research articles, reviews, and clinically relevant information from studies were included based on their relevance to animal venom toxins, mechanisms of action, therapeutic applications, and translational potential.

## 2. Animal Venom Compositions and Major Toxin Classes

Animal venoms are complex biochemical secretions composed of a diverse array of bioactive molecules that can be broadly classified into enzymatic and non-enzymatic components [4,15]. Enzymatic toxins are primarily responsible for venom dissemination into the prey or predators, tissue damage, membrane disruption, inflammation, coagulation disturbances, and modulation of cellular signaling pathways, whereas non-enzymatic toxins can play a role in modulating ion channels, binding to receptors, and exhibiting high target specificity, which can be studied pharmacologically [15].

### 2.1. Enzymatic Toxins Identified Across Venomous Taxa

The adaptive evolution of venom systems has occurred independently across multiple phyla: Cnidaria [2], Arthropoda [16], Mollusca, Chordata, Echinodermata, Annelida, Nemertea, and Nematoda. Each lineage has numerous proteins and peptides that are involved in venomous outcomes [2]. They are present in snakes, snails, insects, spiders, scorpions, cephalopods, centipedes, fish, and even mammals. Common enzymatic toxins that have been identified across venomous taxa, particularly in snakes, bees, scorpions, and spider venoms, are phospholipases A2 [17], metalloproteinases, serine proteases, L-amino acid oxidase, and hyaluronidases. Certain enzyme toxins show taxon-specific distribution, such as Phospholipase D, which is predominantly present in spiders [18], whereas acetylcholinesterases are predominantly found in snake venoms [19]. They are involved in prey digestion and/or direct toxicity, as well as facilitating the spread of toxins into the prey [20].

**Figure 1:**
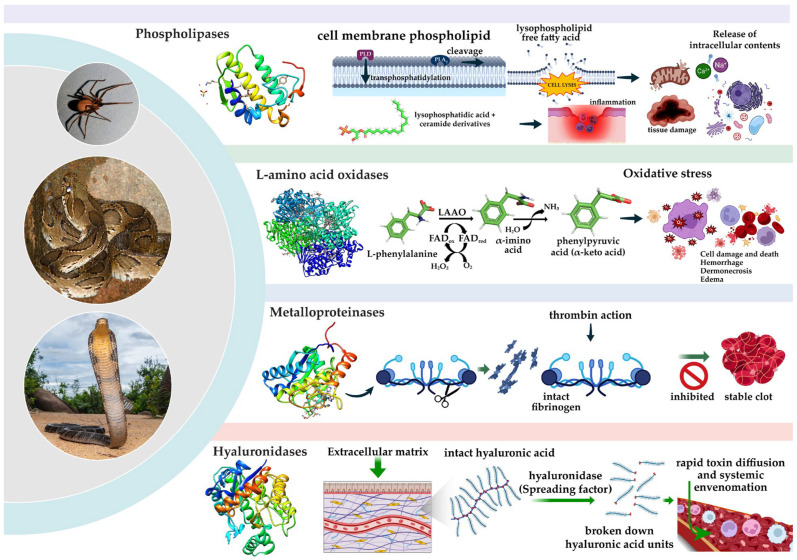
Schematic representation of enzymatic venom toxins: Phospholipases (representative structure: PLA_2_, PDB ID: 1TGM) catalyze the cleavage of membrane phospholipids, which results in membrane permeabilization and disruption of its integrity, causing the release of intracellular contents. Transphosphatidylation reactions via the action of Phospholipase D generate inflammatory mediators such as sphingomyelin-derived metabolites and ceramide derivatives. L-amino acid oxidases (PDB ID: 2IID) cause cytotoxicity by the release of ammonia and hydrogen peroxide. Metalloproteinases (PDB ID: 2W15) hijack the blood clotting system by cleaving fibrinogen, thereby preventing stable clots from forming. Hyaluronic acid cleavage by Hyaluronidase (PDB ID: 1FCQ) causes the breakdown of the extracellular matrix, leading to the diffusion of venom toxins into the bloodstream. (Images of *Loxosceles* sp., *Daboia russelii*, and *Ophiophagus hannah* were adapted from Conway Hawn, sunnyjosef, and Lawrence Hylton, respectively, under CC-BY-4.0 licenses).

Phospholipases (Figure 1) are important venom enzyme toxins that trigger a cascade of toxicity through inflammatory pathways that lead to anticoagulation, cardiorespiratory arrest, edema, myotoxicity, necrosis, and tissue damage [21]. They disrupt membrane integrity by cleaving phospholipids at the sn-2 position of the glycerol backbone, leading to membrane permeabilization and the release of lysophospholipids and free fatty acids as products [21]. In addition, some Phospholipases A2 (PLA_2_) also exhibit neurotoxic activity by affecting presynaptic nerve terminals and disrupting neurotransmitter release, thereby impairing neuromuscular transmission [22]. Other phospholipase families, such as phospholipase D (Figure 1), present in *Loxosceles* spider venom catalyze transphosphatidylation reactions, generating bioactive lipid mediator molecules such as lysophosphatidic acid and derivatives of ceramide, including cyclic versions of it [18,23,24]. These byproducts function as potent signaling molecules that are responsible for inflammatory responses involving leukocyte recruitment and activation, which are amplified, leading to tissue damage [24].

L-amino acid oxidase (LAAO) (Figure 1) is an oxidoreductase enzyme of venom that contributes to envenomation by promoting platelet aggregation, hemorrhagic effects, and induction of apoptosis [25,26]. It is a flavoenzyme that allows the stereospecific catalysis of L-amino acids via oxidative deamination, resulting in the conversion of the amino acid to α-ketoacids, and the production of ammonia and hydrogen peroxide [27]. Predominantly present in snakes, LAAOs are homodimeric proteins that are covalently bound to flavin and bind to a redox-active coenzyme, Flavin Adenine Dinucleotide/Mononucleotide (FAD/FMN) [27]. The generation of hydrogen peroxide during LAAO activity results in reactive oxygen species, which are highly toxic in nature, having the ability to act on proteins, nucleic acids, and cell membranes [28,29]. These Reactive Oxygen Species (ROS) are present extracellularly and act directly on cellular membranes, affecting their permeability, and can induce cell death, like necrosis or apoptosis [30].

Metalloproteinases (Figure 1), abundant in viperid and crotalid venoms, exhibit fibrinolytic activity, activate prothrombin, and induce apoptotic activity in addition to pro-inflammatory effects [31,32]. Envenomation is enhanced with the degradation of extracellular matrix (ECM) proteins such as collagens, fibronectin, vitronectin, laminin, proteoglycans, increasing tissue damage, vascular permeability, and promoting hemorrhage [33].

Hyaluronidase (Figure 1) acts as a “spreading factor” by breaking down a glycosaminoglycan, hyaluronic acid, for the entry and circulation of other toxins into the bloodstream [34,35]. It degrades hyaluronan and chondroitin sulphate [36], by breaking down the glycosidic bonds present in the natural polymer of disaccharide units, resulting in monosaccharide release [37,38,39]. The enzyme is present in several species, including reptiles, arachnids, leeches, caterpillars, hornets, bees, wasps, freshwater stingrays, lizards, mollusks, crustaceans, spiders, and many other organisms, where it results in tissue damage with enhanced venom spread [37].

### 2.2. Non-Enzymatic Toxins

In addition to enzymatic components, animal venoms contain a diverse range of non-enzymatic toxins, including ion channel-modulating peptides, three-finger toxins (3FTxs), conotoxins, Kunitz-type peptides, inhibitor cystine knot (ICK) peptides (knottins), disintegrins, and other receptor-targeting peptides [40,41]. Non-enzymatic venom components also contribute significantly to venom toxicity and pharmacological diversity [15]. These molecules are highly selective and interact with molecular targets such as voltage-gated sodium, potassium, and calcium channels, nicotinic acetylcholine receptors, integrins, and G-protein coupled receptors (GPCR) [41,42]. Through these interactions, non-enzymatic toxins can alter neurotransmission, muscle contraction, platelet aggregation, blood coagulation, and cellular signaling pathways [42]. Ion channel modulators interfere with the activity of ligand-gated or voltage-gated ion channels, resulting in paralysis and altered pain perception [43]. Elapid snake venoms are rich in three-finger toxins, which target nicotinic acetylcholine receptors, inducing neuromuscular paralysis [42,44,45]. Conotoxins from cone snails show remarkable selectivity toward ion channels and neurotransmitter receptors and exert profound effects on neuronal signaling and pain pathways [46,47]. Kunitz-type peptides function mainly as serine protease inhibitors or potassium channel blockers, whereas disintegrins inhibit integrin-mediated cell adhesion and platelet aggregation [48,49]. These toxins are of pharmacological interest due to high target selectivity, making them valuable templates for drug development [6,15]. This class of toxins forms the basis for many venom-derived therapeutics, which are discussed in subsequent sections.

## 3. History’s Milestone: The Discovery of Captopril from an Unusual Source

The relevance of venom-derived components in pharmacology is seen in several drug discovery-related studies, the earliest and most influential being the discovery of Captopril. The discovery of this antihypertensive drug was inspired by bradykinin-potentiating peptides (BPPs) identified in the venom of the *Bothrops jararaca*, the Brazilian pit viper [50]. This landmark achievement represents a breakthrough that revolutionized modern pharmacology and highlights the successful translation of venom-derived molecules into rational drug design and clinical application [51]. Early studies have shown that BPPs inhibit the activity of angiotensin-converting enzyme (ACE), causing a decrease in angiotensin II formation and preventing bradykinin degradation [52]. This dual mechanism provided the foundational framework and the conceptual development of ACE inhibitors, such as captopril [53]. ACE, predominantly expressed in the pulmonary endothelium, plays a key role in the conversion of angiotensin I to II, acting as a potent vasoconstrictor [54]. ACE can also degrade bradykinin, which is a vasodilatory peptide contributing to the regulation of vascular tone [54,55]. Using the findings obtained from this study, Sir John R Vane and colleagues showed the physiological relevance of ACE inhibition, which aided their further attempts at drug discovery [54].

Further, David Cushman and Miguel Ondetti purified a nonapeptide from *B. jararaca* venom, which showed effectiveness in lowering blood pressure but proved to be a poor drug candidate due to a lack of oral bioavailability and its need for being injected into the patient [56]. Later, they modified the peptide based on the action of L-benzylsuccinic acid inhibition of carboxypeptidase A, which is a zinc metallopeptidase, similar to angiotensin converting enzyme peptides [57]. This resulted in the synthesis of a small molecule (succinyl analogues) of venom peptides [57,58]. They eventually made a critical advancement leading to the development of Captopril, which can bind to the ACE active site zinc atom with high potency. Thus, Captopril is a simple dipeptide analogue that has 2000-fold greater inhibition than its predecessor prototypes [56,59]. This groundbreaking discovery paved the way for the management of heart diseases such as heart failure, high blood pressure, [57] as well as other conditions, such as diabetic nephropathy, leading to the Lasker Foundation recognizing Cushman and Ondetti in 1999 with the Albert Lasker Clinical Medical Research Award for their work, identifying them as the pioneers in translational research that bridged venom biology, biochemistry, and clinical therapeutics [59]. This milestone exemplifies how venom-derived molecules, which were once regarded as toxic, life-threatening components, can be transformed into molecular templates for life-saving therapies, highlighting the immense potential of venom-based drug discovery.

## 4. Clinical Applications: Translating Venom-Derived Drugs to the Market

The translation of venom-derived drug candidates represents a major milestone in biomedical research, giving rise to the possibility of several therapeutic applications for cardiovascular diseases, metabolic disorders, blood coagulation, and analgesic properties.

### 4.1. Inhibitors of ACE: Captopril, Enalapril and Quinapril

**Figure 2:**
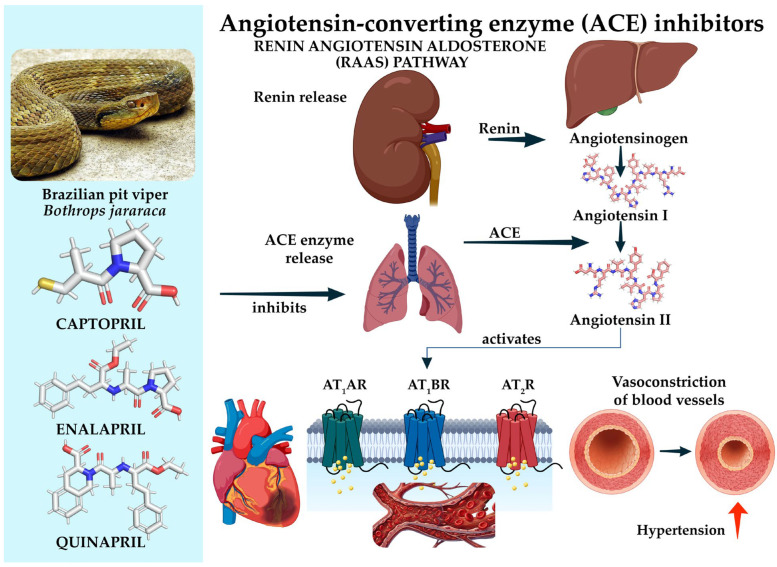
Drugs that inhibit Angiotensin converting enzyme (ACE): Captopril (PubChem CID: 44093), Enalapril (PubChem CID: 5388962), and Quinapril (PubChem CID: 54892), drug derivatives from the venom of the Brazilian pit viper, *Bothrops jararaca*, involved in the inhibition of angiotensin converting enzyme, which prevents angiotensin I (PubChem CID: 3081372) conversion to angiotensin II (PubChem CID: 172198). The binding of angiotensin II to Angiotensin receptors (ATRs) facilitates the vasoconstriction of blood vessels, and when inhibited, it becomes a therapeutic option for hypertension. (Image of *Bothrops jararaca* adapted from Leandro Avelar, licensed under CC-BY-4.0).

Captopril, Enalapril, and Quinapril (Figure 2) are synthetic derivatives of toxins from a common snake species, which can inhibit angiotensin-converting enzyme, commonly used for the treatment of cardiovascular diseases [60]. Captopril (Figure 2) is the first toxin-derived drug to revolutionize the treatment of hypertension [61] and for treating left ventricular dysfunction in patients who have suffered a myocardial infarction [62]. It was developed from bradykinin-potentiating peptides found in *Bothrops jararaca* [63].

Enalapril and quinapril are newer angiotensin-converting enzyme inhibitors that differ from captopril in several key aspects [64]. Unlike enalapril and quinapril, captopril contains a sulfhydryl group in its chemical structure, which may play a role in certain adverse reactions [65]. Additionally, captopril is metabolized directly in the plasma, whereas quinapril and enalapril undergo de-esterification in the liver to form metabolites in their active form, quinaprilat and enalaprilat, respectively [64,66,67].

The Renin–Angiotensin–Aldosterone System (RAAS) pathway is an important regulator of cardiovascular homeostasis, and its dysregulation is associated with hypertension and cardiac disorders [68]. In this pathway, juxtaglomerular cells in the kidney cleave angiotensinogen to form angiotensin I, an inactive precursor [69]. ACE enzyme is located in the vascular endothelium of the kidneys as well as the lungs [70,71,72], and converts angiotensin I to angiotensin II, which is the crucial active peptide of the RAAS pathway [73] (Figure 2). Angiotensin II binds to angiotensin type 1 receptors (AT_1_R; including AT_1_A and AT_1_B subtypes) and type 2 receptors (AT_2_R), functioning as a powerful vasoconstrictor, causing blood pressure to increase [74]. The binding of angiotensin II to AT_1_R triggers a cascade of effects, including inflammation, vasoconstriction, and atherosclerosis, as well as the likelihood of developing insulin resistance and thrombosis [75] (Figure 2). However, on the other hand, binding to AT2R promotes vasodilation, reduction in platelet aggregation, and enhancement of insulin activity [76]. In this system, angiotensin II interacts with the AT1 receptors expressed on smooth muscle cells, leading to vasoconstriction of arterioles and venules at the pre- and post-capillary ends, reducing norepinephrine uptake, and releasing catecholamines from the adrenal medulla, increasing blood pressure [77].

Venom-derived drug peptides inhibit the enzyme that converts angiotensin, known as angiotensin-converting enzyme (ACE), regulating hypertension by preventing vasoconstrictive angiotensin II synthesis [78,79] (Figure 2). Mechanistically, the effectiveness of captopril as a therapeutic intervention is its ability to inhibit the renin–angiotensin–aldosterone pathway by preventing angiotensin conversion [80,81]. By blocking this pathway using venom-derived therapeutics, a reduction in blood pressure takes place due to decreased vasoconstriction [79].

### 4.2. Antithrombotics

Drugs derived from snakes and leeches, Alfimeprase [82], Batroxobin [83,84], Ancrod [85], Bivalirudin, and Desirudin [86], are used to treat blood clotting disorders as well as cardiovascular diseases.

#### 4.2.1. Alfimeprase

**Figure 3:**
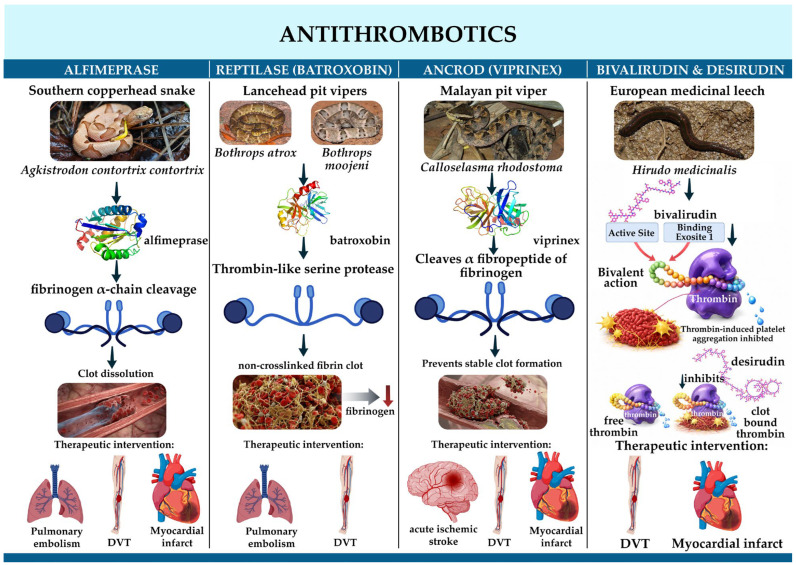
Antithrombotic drugs derived from snake and leech species. Snake venom–derived agents, including Alfimeprase (DrugBank ID: DB04919), a fibrinolytic metalloprotease, and the thrombin-like serine proteases Batroxobin (UniProt: P04971) and Ancrod/Viprinex (UniProt: P47797), target key components of the coagulation cascade, particularly fibrinogen and fibrin, thereby promoting clot dissolution and preventing thrombosis. Leech-derived direct thrombin inhibitors, including Bivalirudin (PubChem CID: 16129704) and Desirudin (PubChem CID: 16129703), inhibit thrombin activity and prevent fibrin clot formation. Together, these biologics target fibrinogen and thrombin to prevent and manage thromboembolic disorders such as pulmonary embolism, deep vein thrombosis (DVT), myocardial infarction, and acute ischemic stroke. (Images of *Agkistrodon contortrix*, *Bothrops atrox*, *Bothrops moojeni*, *Calloselasma rhodostoma*, and *Hirudo medicinalis* were adapted from photographs by Alan Rockefeller (CC-BY-4.0), Whaldener Endo (CC0), Alessandher Piva (CC-BY-4.0), Gerard Chartier (CC-BY-4.0), and Hogler Krisp (CC-BY-3.0), respectively).

Alfimeprase is derived from Agkistrodon contortrix, the southern copperhead snake [82]. This drug is recombinantly produced and is a zinc metalloprotease belonging to the P-1 class of metalloproteinases with an approximate molecular mass of 20–30 kDa, lacking disintegrin-like domains [87]. It has fibrinolytic activity that acts directly on the alpha chain of fibrinogen, with sixfold efficacy compared with certain activators of plasminogen-like urokinase and tissue-type plasminogen activator [88]. The synthesis of fibrinogen takes place in the liver and is converted to fibrin under the action of thrombin during vascular damage [89]. This allows the blood clotting cascade to start to heal the wound; however, when fibrinogen levels are abnormally high in the blood, disease conditions like peripheral vascular disease [90], pulmonary embolism [91], myocardial infarction (Figure 3) [92], stroke, and abdominal aortic aneurysms [93] can arise. Alfimeprase has fast and effective activity against fibrinolysis and prevents a systemic lytic state, unlike usual plasminogen activators [94]. Its unique mechanism of action lies in that it does not require activation of plasminogen, thereby causing a significant reduction in bleeding complications [95]. However, the clinical development of Alfimeprase was stopped after Phase III trials (NAPA-2, SONOMA-2) as the results did not show a higher efficacy over existing treatment for patients suffering from peripheral arterial occlusion (PAO) and showed to cause increasing rates of hemorrhage and embolisms [96]. Although studies done previously showed rapid action in catheter clearance, the main purpose of the drug endpoint, which is avoiding surgical intervention, failed [96,97]. Despite this, Alfimeprase has received the status of an Orphan drug for the treatment of patients with ST-elevation myocardial infarction (STEMI) [98].

#### 4.2.2. Reptilase (Batroxobin)

Reptilase is a serine protease that is thrombin-like, obtained from the venoms of two medically significant pit vipers found in South America, the *Bothrops atrox* and *Bothrops moojeni* [83,84]. There are 12 cysteine amino acid residues, which together make up 231 amino acids, forming six disulphide bonds [99]. Batroxobin cleaves the α and β chains of fibrinogen, resulting in the formation of non-crosslinked fibrin clots by targeting Arg16-Gly17 residues [100,101]. Batroxobin is used for blood-clotting disorders, including pulmonary embolism [102] and deep vein thrombosis [103] by reducing fibrinogen levels in the blood through fibrinolysis (Figure 3). Currently, it is being used medically for diagnostic purposes as an in vitro reagent, but has limited use in clinical settings [104]. One of the main reasons for its usage in diagnostic settings as an in vitro agent is because of Heparin insensitivity [105]. Batroxobin cleaves fibrinogen into fibrin irrespective of the presence of heparin and does not get inhibited, permitting an accurate measurement of fibrinogen levels, which can be used to assess coagulation disorders [106]. Due to its specificity towards fibrinopeptide A, it also aids in the diagnosis of congenital and acquired fibrinogen defects without the interference of other clotting factors present in the sample [83,105,106]. Additionally, well-established anticoagulant and thrombolytic agents are available, which could override the need to use Batroxobin clinically. The production of standardized preparations from snake venom has proven to be challenging, as the enzyme is present in low concentrations and requires complex purification [5]. However, these challenges have prompted the development of other recombinant alternatives to provide a scalable and consistent source of batroxobin [101,107].

#### 4.2.3. Ancrod (Viprinex)

Possessing coagulant, esterolytic, and proteolytic properties, Ancrod, a thrombin-like serine protease isolated from the Malayan pit viper (*Calloselasma rhodostoma*) [85], is a glycosylated protein having a molecular mass of approximately 28–32 kDa, used mostly in a recombinant form [108] and acts with a defibrinogenation mechanism of action [85,100,109]. They act by having a proteolytic effect on circulating fibrinogen by cleaving the fibrinogen α chain without cleavage of the β chain, facilitating its rapid clearance, resulting in low fibrinogen levels and decreased blood viscosity [110,111]. Ancrod is used for the treatment of myocardial infarction, deep vein thrombosis, acute ischemic stroke, and priapism (Figure 3) [112,113].

#### 4.2.4. Bivalirudin and Desirudin

Both Bivalirudin and Desirudin are recombinant derivatives of hirudin, which is an acidic peptide obtained from the salivary glands of the European medicinal leech, *Hirudo medicinalis* [86]. Bivalirudin is a 20-amino acid peptide and is derived as an analogue of a natural anticoagulant molecule from the salivary glands of the European medicinal leech, *Hirudo medicinalis* [114]. It has a molecular mass of 2.18 kDa and is used for treating deep vein thrombosis and coronary angioplasty repair [115,116] due to its anticoagulant properties [114]. It acts via the activation of two distinct sites with a direct bivalency action onto thrombin. It binds to the active site as well as the binding exosite 1 present on fibrinogen [115,117]. Additionally, it also inhibits clot-bound thrombin (Figure 3), as well as having an anti-platelet effect by inhibiting thrombin-induced platelet aggregation [118].

Desirudin is a 65-amino acid polypeptide with a molecular mass of approximately 7 kDa and is stabilized by disulphide bonds [119]. It is similar in action to Bivalirudin, which is a direct mechanism of inhibition acting on free and fibrin-bound thrombin, used effectively as therapy for deep vein thrombosis (DVT) by preventing clotting factors V, VIII, and XIII activation [119]. Desirudin is a non-heparin subcutaneous drug that has shown more efficacy than unfractionated heparin and enoxaparin, and has shown significant changes in bleeding rates for preventing DVT in patients who have hip replacement surgery [119]. It has also proved to be advantageous by being less immunogenic than unfractionated heparin [119].

### 4.3. Antiplatelet Action

Derived from snakes, antiplatelet drugs are not only used for effective treatment of blood-clotting disorders but also for ischemic coronary conditions. They act by targeting platelet glycoprotein IIb/IIIa receptors (integrin alpha IIb/beta 3), facilitating an inhibitory action of fibrinogen binding, preventing platelet aggregation, which ultimately reduces thrombus formation [120]. Consequently, due to this therapeutic effect, they are effective in treating blood-clotting disorders and preventing thrombus formation during percutaneous coronary interventions.

#### 4.3.1. Aggrastat

**Figure 4:**
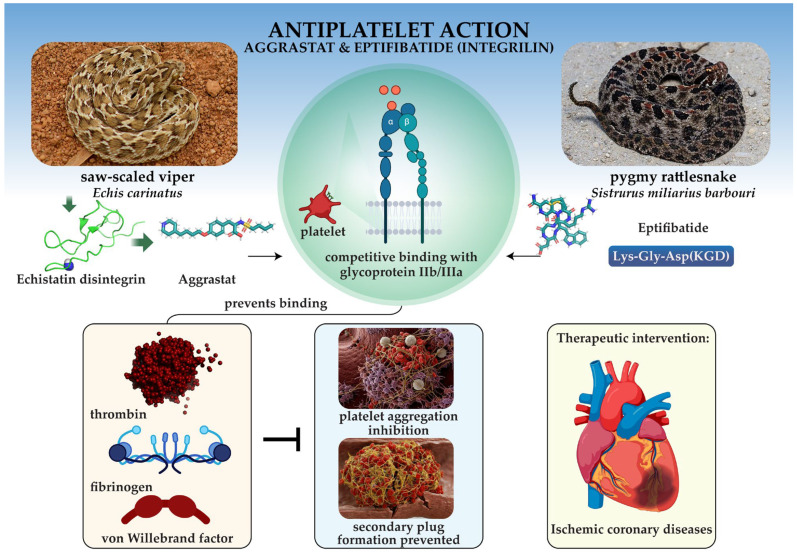
Antiplatelet action of drugs, Echistatin disintegrin, (PDB ID: 2ECH), Aggrastat (PubChem CID: 60947) and Eptifibatide (PubChem CID: 448812), derived from snakes and used to treat coronary conditions: Snake venom inspired antiplatelet drugs target glycoprotein IIb/IIIa receptors to inhibit fibrinogen-mediated platelet aggregation and thrombus formation to prevent diseases like pulmonary embolism, deep vein thrombosis (DVT), Myocardial infarct, and acute ischemic stroke. (Images of *Echis carinatus* and *Sistrurus miliarius barbouri* adapted from Hopeland and Laura Gaudette, under CC-BY-4.0, respectively).

Aggrastat or Tirofiban (Figure 4) is synthetic non-peptide small molecule antagonist of the receptor platelet glycoprotein IIb/IIIa (integrin alpha IIb/beta 3) that is designed based on the RGD (Arginine, Glycine and Aspartic acid) tripeptide motif rationale present in echistatin, isolated from the venom of *Echis carinatus*, or the saw-scaled viper [121,122]. Echistatin is a cysteine-rich low molecular mass (~5 kDa) protein from the disintegrin family, and its structure is stabilized by multiple disulphide linkages and aids its high affinity towards integrin receptors, including αIIbβ3, αvβ3, and α5β1 [120]. It is important to note that Tirofiban is not a direct venom derivative and is a synthetic peptidomimetic based on the integrin-binding motif and disintegrin structure [123]. It is highly specific to glycoprotein receptors IIb/IIIa, which are highly expressed on platelets, preventing the binding of von Willebrand factor and fibrinogen, and preventing thrombus formation [124].

#### 4.3.2. Eptifibatide (Integrilin)

Eptifibatide or Integrilin (Figure 4) is a synthetic cyclic heptapeptide of molecular mass 832Da and is derived from barbourin, which is a disintegrin molecule characterized from *Sistrurus miliarius barbouri*, the pygmy rattlesnake [125,126] It inhibits glycoprotein IIb/IIIb (integrin alpha IIb/beta 3) present on the surface of platelets by reversible binding [125,126,127]. This toxin binds to integrin alpha IIb/beta 3, preventing platelet activation, platelet adhesion to fibrinogen and other ECM molecules, as well as platelet aggregation, and consequently thrombus formation [128]. Eptifibatide is prescribed as part of treatment against ischemic coronary conditions because of its binding to glycoprotein integrin alpha IIb/beta 3, due to a peptide sequence KGD that mimics the RGD sequence in the ECM components involved in platelet activation, adhesion, and aggregation, leading to competitive binding, preventing fibrinogen and von Willebrand factor from binding to platelets [128].

### 4.4. Metabolic Disorders: Exenatide (Byetta)

The Gila monster (*Heloderma suspectum*) venom has peptide derivatives that are effective in treating metabolic disorders and lifestyle conditions like obesity [129]. Targeting specific receptors in the pancreas that have an impact on diabetes makes it an appealing therapeutic intervention [130].

**Figure 5:**
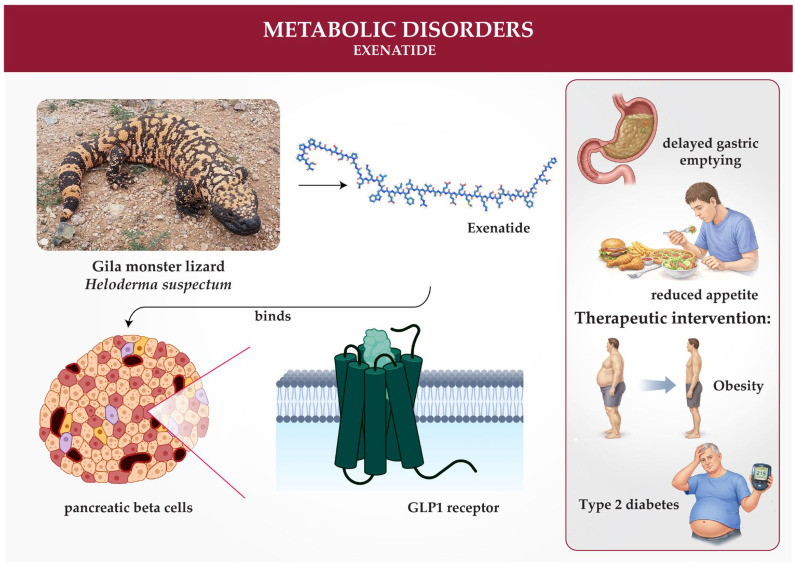
Synthetic peptide derivatives Exenatide (PubChem CID: 45588096) from the Gila monster lizard to treat metabolic disorders: Regulate glucose dysfunction by binding to receptors on pancreatic beta cells and by reducing obesity through delayed gastric emptying. (Image of *Heloderma suspectum* adapted from Josh Olander, CC-BY-4.0).

Exendin-4 (Figure 5) is isolated from the Gila monster lizard (*Heloderma suspectum*) and is 4.2 kDa in size, a 39-amino acid peptide molecule, which has approximately 53% similarity to GLP1 (glucagon-like peptide 1) [130]. It consists of a critical N-terminal sequence, which is required for activating GLP1, and the difference in sequences enables it to be resistant to DPP4-mediated degradation, making it stable with prolonged activity [131]. Exendin-4 is predominantly an α-helical structure with C-terminal differences that distinguish it from native GLP-1, hence its prolonged pharmacokinetic stability [132]. Its synthetic peptide analog, Exenatide, is used, which is structurally similar to Exendin-4, and is used as a therapeutic intervention for Diabetes mellitus (Type 2) [133]. It binds to the GLP-1 receptor (GLP-1R) present on the surface of pancreatic beta cells and increases the release of insulin, depending on glucose levels [134,135]. This additionally affects gastric emptying by delaying it due to a reduction in food intake [136,137]. This aids the process of weight loss in patients with type 2 diabetes who are also struggling with obesity [138].

Exenatide is a receptor agonist that generally has several other advantages other than glycemic control and weight reduction, including obesity associated complications, cardiovascular disease, insulin resistance, blood pressure control, and metabolic syndrome [139,140,141]. By improving glucose homeostasis and promoting sustained weight loss, exenatide contributes to improved cardiometabolic health and may help reduce the long-term risk of diabetes-related complications and obesity-associated morbidity [142].

Discovering Exendin 4 from *Heloderma suspectum* venom represents one of the most successful examples of venom-derived drug development [143]. The successful translation of Exendin-4 into Exenatide established the foundation for the modern GLP-1 receptor agonist class, demonstrating a transformative impact on the treatment of type 2 diabetes and obesity [144].

### 4.5. Analgesics: Cobratide and Ziconotide (Prialt)

Chronic pain is a serious health issue, and promising drug molecules have been isolated and characterized from cobra and cone snails, which not only have analgesic properties but have also shown effectiveness in viral infections like COVID-19 [145,146].

**Figure 6:**
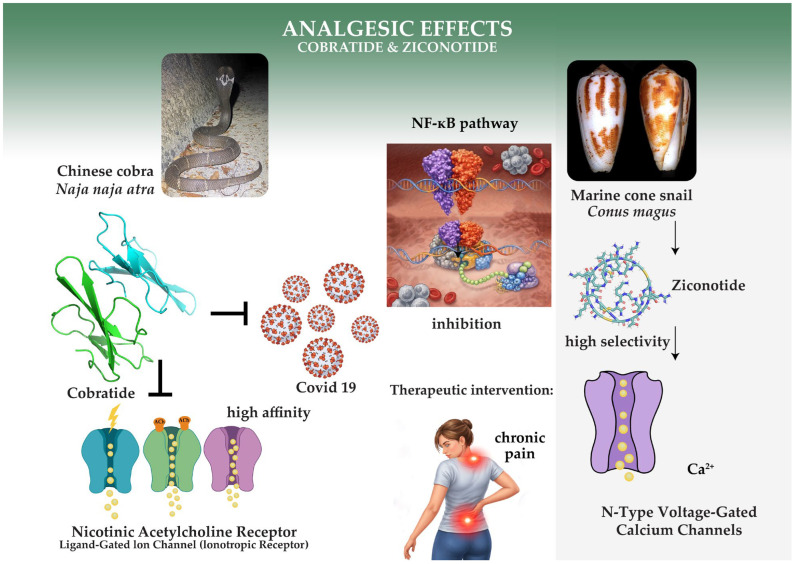
Peptide derivative drugs Cobratide (PDB ID: 1V6P) and Ziconotide (PubChem CID: 16135415) from cobra and snail species, diverse disease conditions: Shown inhibition in COVID-19, inflammatory pathways, and high affinity and selectivity towards ion channel receptors, providing a therapeutic strategy for chronic pain and nociception. (Images of *Naja naja atra* and *Conus magus* were adapted from Lawrence Hylton (CC-BY-4.0) and Richard Parker (CC-BY-2.0), respectively).

Cobratide (Figure 6) is an analgesic peptide for chronic pain derived from the α-cobrotoxin from *Naja naja atra* (Chinese cobra) [147], and also proved to be an ideal therapeutic intervention for COVID-19 [145,148]. It belongs to the three-finger toxin (3FTx) family and is stabilized by 62 amino acid residues in length and 4 disulphide bonds, having a low molecular mass of approximately 7 KDa [149]. α-cobrotoxin adopts a three-finger loop structure that has high binding affinity to nAChRs (acetylcholine receptors) present in the neuromuscular junction, hence modulating ion channel activity and neuronal signaling [150]. Additionally, studies have shown that the cobrotoxin can inhibit the NF-κB pathway, which is an important pathway in inflammatory conditions, exerting an anti-inflammatory effect [151], hence bringing down the cytokine storm that damages the lungs during COVID-19 infection [145].

Ziconotide (Figure 6) ω-MVIIA, an omega-conotoxin, is a synthetic form of a 25-amino acid polypeptide (~2.6 kDa) present in the venom of the fish-eating marine snail, *Conus magus* [152]. It’s part of the inhibitor cystine knot (ICK) peptide family, and it is characterized by disulphide linkages between six cysteine residues that ensure proper peptide folding and structural rigidity, making it resistant to proteolytic degradation [153]. Ziconotide exhibits high selectivity for CaV2.2 (N-type voltage-gated calcium channels), which are predominantly expressed at the presynaptic terminals of primary afferent nociceptive neurons in the dorsal horn of the spinal cord [154]. It is administered intrathecally for the treatment of chronic pain [146]. During pain transmission, action potentials are generated and propagated along nociceptive neurons by voltage-gated sodium channels, which trigger the opening of presynaptic N-type calcium channels [155]. The resulting calcium influx promotes the release of excitatory neurotransmitters such as glutamate, substance P, and Calcitonin Gene-Related Peptide (CGRP) [156]. Under normal physiological conditions, nociceptive signaling is facilitated. However, this becomes a dysregulated process during chronic pain. Ziconotide selectively blocks CaV2.2 (N-type) calcium channels and reduces calcium-dependent neurotransmitter release, interrupting nociceptive signal transmission, producing analgesic effects [157,158,159].

## 5. Potential Drug Targets of Animal Venoms

Compounds derived from animal venoms can be organized broadly into two categories: clinically approved therapeutic interventions and bioactive molecules or peptides with considerable pharmacological and biotechnological potential that have not yet undergone clinical evaluation and been translated into approved drugs [160]. While some venom-derived molecules and analogues have been integrated into medical applications, a significant number of toxins remain under inquiry due to issues with efficacy and safety [160]. This section emphasizes the emerging toxin candidates that target ion channel receptors and enzymes, blood coagulation pathways, and represent promising leads for drug development for different diseases. Table 1 provides an overview of the principal molecular targets, representative animal toxins, mechanisms of action, and associated therapeutic applications discussed in this section. It is organized in a species-specific manner to elucidate the taxonomic diversity of venom-derived molecules and to emphasize the comparison of toxin classes, molecular targets, and therapeutic applications across different venomous taxa.

### 5.1. Ion Channel Targets

Ion channels represent an important class of therapeutic targets due to their role in regulating neuronal excitability, muscle contraction, and signal transduction [197,198]. Dysregulation of ion channels is associated with several pathological conditions, including chronic pain, and cardiovascular disorders [199,200]. Venom-derived molecules serve as valuable pharmacological tools for therapeutic intervention by selectively targeting these systems [201].

Among venom-derived molecules targeting ion channels, cardiotoxins from snake venom are members of the three-finger toxin family with a low molecular mass typically ranging from 6 to 8 kDa, and have cardioprotective or cardiotoxic effects [202,203]. These toxins act on the heart muscles, the smooth muscles of the vascular system, and the vascular capillary bed [203].

Ion channel modulation is also mediated by neurotoxins, including enzymes such as secretory phospholipases (PLA_2_), which function as presynaptic neurotoxins, along with peptides such as three-finger toxins, kunitz-type serine protease inhibitors (dendrotoxins), cysteine-rich secretory proteins, and serine proteases that have a unique ability to modulate voltage-gated potassium channels (Kv) [204].

By targeting nAChRs (postsynaptic nicotinic acetylcholine receptors), α-neurotoxins known as Curaremimetic (Table 1), derived from elapid and hydrophiid snakes, are highly specific and selective toward nAChRs, thereby inhibiting neurotransmission of acetylcholine at the neuromuscular junctions of skeletal muscles [44,205]. These toxins are short-chain and approximately 62 residues, whereas the long chains are approximately 74 residues in length, with both having 4 and 5 disulphide bridges, respectively [206]. Binding of acetylcholine to nAChRs opens ligand-gated cation channels, allowing sodium influx and membrane depolarization, which subsequently initiates muscle contraction under normal physiological conditions. Prevention of acetylcholine binding by receptor blocking by α-neurotoxins prevents neuromuscular transmission, resulting in muscle paralysis [44,205]. α-neurotoxins have high specificity to nAChRs, [207] and this makes them clearly distinguished from the muscarinic receptor antagonists atropine and scopolamine [208], making them valuable pharmacological tools for investigating neuromuscular transmission and receptor structure–function relationships.

α-Bungarotoxin (Table 1), present in *Bungarus multicinctus*, has high specificity towards nAChR α1 due to the presence of arginine (36) and phenylalanine (32) amino acids in the finger II present in the toxin, which can block the receptor at the aromatic cage [162]. This also leads to the disruption of the interaction between the Cys-loop glycan chain and the C lip of nAChR, which blocks communication between the membrane pore and the ligand binding site [209]. Additionally, disruption of the glycan chain in the cysteine loop and C loop takes place, which prevents the ligand binding site and membrane pore from communicating with each other [206,209].

Acid-sensing ion channels (ASICs) are an important class of targets that are involved in signaling and neuronal injury [210,211]. Mambalgins (Table 1), 57-amino acid-long, acidic polypeptides that are isolated from the black mamba venom, inhibit ASICs and exhibit analgesic effects comparable to morphine [168,169,170]. The toxin has a functional domain that inhibits the acid-sensing ion channel, which is voltage-dependent and is activated by acidification using the extracellular milieu [170]. They are homotrimeric/heterotrimeric structures that are activated by protonation in the extracellular environment, which takes place during neurotransmission, inflammation, and ischemia, causing excitatory sodium influx in pain signalling [212]. They have been recognized in several pathophysiological conditions, ranging from neuronal injury, nociception, mechanoperception, and synaptic plasticity, and hence are important pharmacological targets for pain management, psychiatric disorders, neurodegenerative disorders, and stroke [170].

Voltage-gated sodium channel NaV1.7 plays a crucial role in pain signalling [173]. A short-chain neurotoxin called Calliotoxin (δ-elapitoxin-Cb1a) (Table 1), characterized and isolated from the coral snake, *Calliophis bivirgatus*, modulates voltage-gated sodium channels and influences neuronal excitability [172]. Other sodium channel inhibitors include µ-TRTX-Hhn2b (HNTX-I), (Table 1) is a 33-residue length peptide isolated from *Selenocosmia hainana*, and Huwentoxin-IV of 35 amino acid length (HWTX-IV) (Table 1) from *Ornithoctonus huwena* [181]. µ-TRTX-Hhn2b (HNTX-I), has 3 sulphide bridges and forms a cystine knot that inhibits Na_V_1.7 in pain signalling and the regulation of sensory neuronal excitability, and is important in various sensory mechanisms [173]. Huwentoxin-IV (HWTX-IV), also a member of the ICK (Inhibitory cystine knot) family, is shown to reduce neuropathic pain as reported in animal model studies via the inhibition of NaV1.7 in chronic pain, which acts by amplifying a stimulus, causing the initiation of an action potential in nociceptors [213]. Similarly, µ-TRTX-HI1a (Table 1) is a newly detected 39 amino acid long neurotoxin from *Haplopelma lividum* spider that is responsible for the inhibitory action of sodium channels, Na_V_1.8, providing analgesic properties [180,214].

Conotoxins, derived from Conus species, are disulfide-rich peptides with selective modulation of ion channels and receptors present in neuronal and muscular function [196]. They act by targeting voltage-gated sodium (NaV), potassium (Kv), and calcium channels (CaV) [215,216]. This influences key physiological processes such as the generation of action potential, the release of neurotransmitters, and transmission through synapses [195]. GIIIA (*Conus geographus*) (Table 1) and PIIIA (*Conus purpurascens*) (Table 1) are µ-conotoxins that block voltage-gated sodium channels, which can prevent neuropathic and inflammatory pain [194]. PVIA (Table 1) is a δ-conotoxins (*Conus purpurascens*) that also targets the same sodium channels and is potentially a promising therapeutic for epilepsy [195]. Contrastingly, PVIIA (Table 1) is a κ-conotoxin *(Conus purpurascens*) that acts on voltage-gated calcium channels, influencing neuronal excitation [196].

Voltage-gated calcium channels are important for the generation of electrical signals in neuronal cells, as well as muscle and cardiac tissue, which require excitation [171]. Acting as a smooth muscle relaxant, Calciseptine, a 60-amino acid peptide isolated from the black mamba *Dendroaspis polylepis* (Table 1), reduces contractions taking place in cardiac tissues [171]. It is a strong L-type calcium channel blocker that prevents calcium from entering the channel by binding to the 1,4-dihydropyridine binding site [171]. In addition, Calciseptine is not active against N and T types voltage-dependent calcium channels and has specificity for only L-type calcium channels [171].

Spider venoms are highly specialized venoms that represent a rich source of ion channel modulators [180,217]. Australian funnel-web spiders (*Atrax robustus* and *Hadronyche versuta*) have peptides in large numbers that range from 2 to 8 kDa in molecular mass, and their analysis showed that a majority of their peptides, which are already characterized, showed a high affinity to ion channels and their subtypes [180,217]. Disulphide-rich peptides modulate acidic ion sensing, mechanosensitive channels, and channels activated by calcium and potassium ions. They also modulate voltage-gated potassium and sodium channels, transporters, and glutamate receptors [180].

Mechanosensitive channels are targeted by GsMTx4 (Table 1), a toxin isolated from the tarantula, *Grammostola spatulata*, which can specifically target Piezo, a mechanosensitive channel, which reduced pulmonary hypertension in mice [180].

PhTx3 (Table 1) from spider venom of *Phoneutria nigriventer* acts on voltage-dependent calcium channels (CaV) [218]. It can inhibit channels of type N, R, and P/Q and has been preclinically proven to have a pain-relieving effect in several studies [219,220] by specifically binding and inhibiting the type N channel, reducing glutamate neurotransmitter release in the spinal cord [221].

Potassium channels (Kv) are important therapeutic targets in immunological modulation and are targeted by ShK toxin isolated from *Stichodactyla helianthus*, a Caribbean Sea anemone [222]. Activated effector memory T cells express Kv1.3, which plays a crucial role in the maintenance of the membrane potential required for calcium signaling during T cell activation [223]. By blocking Kv1.3 channels, potassium efflux is reduced, thereby suppressing T-cell activation, proliferation, and pro-inflammatory cytokine release, making these channels attractive therapeutic targets for autoimmune disorders [215,216]. Studies using molecular modelling revealed that Lys22 is responsible for blocking the potassium channel by penetrating the pore and occluding it. The other amino acids, such as Serine (20th), Lysine (25th), and Tyrosine (23rd), help the toxin to bind to the voltage-gated channel as seen in animal models [222]. Dalazatide (Table 1) is an analogue of the ShK peptide toxin, which has also been studied for disorders of autoimmune nature, specifically, polyangiitis, granulomatosis [182], and plaque psoriasis [213].

Scorpion venoms consist of a large group of peptides of neurotoxic nature that target ion channels, especially potassium channel toxins (KTxs), and they behave in a highly selective manner [224]. They are small peptides of 20 to 40 amino acids in length and form a conserved cystine-stabilized scaffold (α/β (CS-α/β) scaffold) with high structural stability and specificity towards ion channels [225]. KTxs bind selectively to voltage-gated potassium channels (Kv) and K^+^ channels activated by calcium (KCa) by occluding the pore and preventing the efflux of K^+^ ions [224,226]. This results in the impairment of membrane repolarization and prolongs depolarization [224,226]. Charybdotoxin (ChTX) (Table 1) is a well-characterized 37-amino acid peptide (~4.3 kDa) from scorpion venom of *Leiurus quinquestriatus*, known as the “Deathstalker”. It consists of three disulphide bonds and an N-terminus that is modified with pyroglutamate, blocking the potassium channels at nanomolar concentrations with a highly specific interaction [188]. Iberiotoxin (IbTX) (Table 1) (*Buthus tamulus*) has 68% sequence homology to ChTX and functions by blocking large conductance calcium-activated potassium channels [189]. Margatoxin (MgTx) (Table 1) from *Centruroides margaritatus* [190] and Vm24 (Table 1) from *Vaejovis mexicanus smithi* are Kv1.3 channel blockers, which are potent and specifically involved in T-cell regulation [191,192]. This helps in blocking its activation, proliferation, and release of proinflammatory cytokines, making it a promising therapeutic intervention for autoimmune disorders [190,227] such as Multiple sclerosis, psoriasis, and rheumatoid arthritis [227]. Collectively, KTxs are potential ion channel blockers that can have a therapeutic role in autoimmune, cardiovascular, and neurological disorders [190,227,228].

### 5.2. Receptors and Enzyme Inhibition

Receptors and enzyme systems are crucial therapeutic targets due to their central roles in general physiological functions [229,230,231]. Venom-derived components exhibit high specificity towards these targets, making them useful templates for drug discovery and pharmacological intervention [232].

Adrenergic receptors are key targets in cardiovascular regulation as they modulate heart rate, vascular tone, and blood pressure [202,233]. Specific three-finger toxins, such as β-cardiotoxins from king cobra (*Ophiophagus hannah*), function as potent beta-blocker-like molecules by targeting β_1_ and β_2_ adrenergic receptors [234]. In addition, ρ-toxins from the green mamba (*Dendroaspis angusticeps*) interact with α-adrenergic receptors, highlighting their potential as highly selective templates for developing therapeutics against hypertension and heart failure [202,235].

Cardioprotective effects from venom-derived components such as lebetin, a natriuretic-like peptide identified from *Macrovipera lebetina* venom, demonstrate protective effects on the heart post myocardial infarction [176,177]. Using in silico methods, the mechanisms of cardioprotective effects were understood, and the studies showed affinity for all human natriuretic peptide receptors, suggesting a potential treatment for decompensated heart failure [236].

Nicotinic acetylcholine receptors (nAChRs) are the main targets in neuromuscular transmission and neuronal signalling [237]. Long-chain α-neurotoxins from snake venom are a subgroup of the three-finger toxin superfamily with a conserved structural fold where three β-stranded loops extend from a hydrophobic core [238]. Typically, they are approximately 74 amino acid residues in length and are stabilized by five disulphide bonds [239,240]. They are specific towards neuronal nAChR, which makes them ideal drug candidates for Parkinson’s disease, while also being a molecular probe as a potential analgesic [206]. Additionally, it is also an anti-inflammatory molecule and immunosuppressant in nature, as the toxin isolated from *Naja atra*, the Chinese cobra, nullified allograft rejection in rats by inhibiting a T-cell-mediated response [206]. Further modulation of nAChR is observed with Ω-neurotoxins, Oh9-1, isolated from the King Cobra (*Ophiophagus hannah*), which interact with rat αβεδ and α3β2 nAChR [167]. κ-bungarotoxins—they are similar to α-neurotoxins, existing in a dimeric conformation and specifically recognizing α_3_β_2_ and α_4_β_2_ nAChR subtypes [164,165,166]. Similarly, Haditoxin is a homodimeric three-finger peptide toxin isolated from the king cobra *O. hannah* and is a structural homolog to curaremimetic short-chain α-neurotoxins, curaremimetic peptides, and they specifically interact with neuronal α7, α3β2, and α4β2 nAChRs with high affinity towards α7, as well as with muscle nAChRs (α1βδε) [163].

Muscarinic toxins bind to muscarinic acetylcholine receptors (mAChRs), which are GPCRs on the surface of neurons [241] and can behave antagonistically or agonistically towards mAChR (M1-M5). Muscarinic toxin from *Naja kaouthia* binds agonistically to M1 and antagonistically to M4 [242,243]. This makes it ideal for distinct biomedical investigations and can be a potential target for neuropsychiatric conditions like schizophrenia or neurodegenerative disorders like Alzheimer’s and Parkinson’s disease. Venom toxins from the green mamba (*Dendroaspis angusticeps*) MT3 and MT7 have high specificity for M4 and M1 mAChRs, respectively [242].

### 5.3. Hemostatic Targets

The coagulation cascade and related proteolytic pathways involve important targets in regulating thrombosis, fibrinolysis, and hemostasis [244,245,246]. Circulating blood cells, coagulation factors, and vascular wall components of mammalian species are considered targets of snake toxins [247]. Venom-derived molecules have high specificity towards key enzymes and cell surface receptors in these pathways, which makes them promising candidates in the development of antithrombotic, anticoagulant, and hemostatic agents [248,249,250].

Serine proteases in the blood clotting cascade are targets of a potent peptide such as Poecistasin (Table 1), present in the leech *Poecilobdella manillensis* [179]. It is a 48-amino-acid protein, specifically targeting serine proteases involved in coagulation and inflammation, particularly factor XIIa and kallikrein present in the intrinsic coagulation pathway, exerting anti-thrombotic effects [183,184].

Similarly, antistasin (Table 1) is another serine protease inhibitor, 119 residues long, isolated from *Haementeria officinalis*, which inhibits Factor Xa and elicits an anticoagulation effect [185]. Another inhibitor of Factor Xa, ghilanthen (Table 1), isolated from the salivary glands of *Haementeria ghilianii*, prolongs prothrombin time, contributing to anticoagulation [251]. On the other hand, Hirustasin and Bdellastatin (Table 1), obtained from the venom of *Hirudo medicinalis*, inhibit tissue kallikrein without inhibiting factor Xa, indicating a distinct mechanism of modulating coagulation pathways [186,187].

Integrins are an important target in the clotting pathway and play a crucial role in platelet aggregation and cell adhesion [252]. Dendroaspin (Table 1) is a venom protein with disintegrin-like properties isolated from the Jameson’s mamba (*Dendroaspis jamsonii*), which interacts with αIIbβ3, *α*_V_β_3,_ and α_5_β_1_ at the extracellular domain [178,179]. There is an Arg-Gly-Asp (RGD) tripeptide sequence that exhibits binding to integrins and is a component of Dendroaspin [253]. Dendroaspin is representative of elapid venoms, and such integrin targeting activity is more associated with disintegrins and snake venom P2-P3 metalloproteinases, found in Viperidae venoms [254]. This RGD motif facilitates competitive binding to integrins, thereby preventing their interaction with ECM proteins and platelet ligands, resulting in inhibition of platelet aggregation and cell adhesion; thus, Dendroaspin exhibits a mechanism analogous to classical disintegrins despite its evolutionary origin [168,169,170].

Fibrinolytic pathways are also important targets for regulating clot stability [246]. Two isoforms, Textilinin-1 and Textilinin-2 (Table 1), purified from the Eastern brown snake, *Pseudonaja textilis*, differing in 6 amino acids, have shown in studies using murine tail vein models, reduced 60% of bleeding via the gradual and tight binding and inhibition of plasmin [174,175]. They are Kunitz-type serine protease inhibitors that are structurally homologous to Aprotinin (Table 1), used as a hemostatic therapy in cardiac surgery to control bleeding [255]. It is 60 amino acids long with three conserved disulphide bridges and specifically acts on plasmin and kallikrein, which are serine proteases, stabilizing clots and reducing the risk of excessive bleeding by inhibiting fibrinolysis [256,257,258].

## 6. Animal Venom Toxins with Pharmacological Potential Based on Their Activities

### 6.1. Anti-Cancer Therapeutics

Cancer is a global health challenge that continues to require novel therapeutic agents due to the ability of the disease to evolve resistance, adapt survival strategies, and recur in a patient’s lifetime [259]. Due to the heterogeneous nature of the disease, it is important to consider all possible drug sources, including venom-derived anti-cancer agents to induce apoptosis, inhibit tumor progression, and disrupt cancer cell membranes [260,261,262]. Venom-derived anti-cancer agents contain several toxin families that structurally and mechanistically differ from each other. Cytotoxins I and II from *Naja oxiana* showed cytotoxic and anti-proliferative activity with better activity than the anti-cancer drug cisplatin against breast cancer, as well as cytotoxicity towards glioblastoma multiforme by promoting apoptotic cell death through intrinsic mitochondrial and ROS-mediated cell death [193,263,264,265]. Apoptotic activity was seen in cancerous cell lines for liver cancer (HepG2), breast cancer (MCF-7), acute promyelocytic leukemia (HL-60), and prostate cancer (DU-145) by the cytosolic release of cathepsins or via the lysosomal pathway [193].

Arthropod venoms also have well-characterized anti-cancer peptides like chlorotoxin (Table 1) from *Leiurus quinquestriatus* (scorpion), which have shown promising results with their high specificity to glioma and have proceeded to advance clinical evaluation, highlighting their translational relevance from bench to bedside [193]. Anti-cancer potential has also been observed in spider-venom peptides by suppressing tumor progression, induction of necrotic cell death, prevention of cell migration, and pro-apoptotic activity, disrupting tumor cell membranes [266]. Brachyin, a neurotoxin obtained from *Brachypelma albopilosum*, has prevented cell proliferation in metastatic melanoma cell line (C8166), T-cell leukemia (Molt-4), lung carcinoma (A549), human bladder cancer cells (BIU-87, T24), and human lung adenocarcinoma cells (Calu-6) [266,267]. Lycosin is another anti-cancer peptide from *Lycosa singorensis* venom that can induce apoptosis extrinsically via the mitochondrial cell death pathway [266]. It promotes cancer cells to undergo apoptosis and also causes the upregulation of p27 (a cyclin-dependent kinase inhibitor), inhibiting cellular proliferation [266].

Apart from these examples, Melittin is a bee venom peptide that has shown the ability to disrupt cell membranes and promote apoptotic cell death in cancer cells [268,269]. Additionally, mastoparan from wasp venom has been shown to have significant anti-cancer properties [270] especially in breast cancer [271], lung cancer [272] and melanoma [273].

### 6.2. Antimicrobial Therapeutics

Antibiotic resistance remains a burgeoning global threat, and the need for new antimicrobial drugs is imminent [274,275]. Snake venom phospholipases are useful antimicrobial candidates due to their broad-spectrum action, exerting their effects on the bacterial membrane [275]. PLA_2_s bind to negatively charged cell membranes of the bacteria, leading to an increase in permeability, formation of pores, and eventually lysis of the bacterial cell [275]. Secretory phospholipases (PLA_2_) isolated from a pit viper, *Protobothrops mucrosquamatus*, were checked for antimicrobial effects against Gram-positive and Gram-negative strains and showed that they were able to inhibit *Pseudomonas aeruginosa*, a bacterium that can survive in immunocompromised patients, causing infections, and *Salmonella typhimurium*, which causes typhoid fever [276,277]. Studies of a novel phospholipase A_2_ from the *Bungarus faciatus* snake known as BFPA have also shown potent bactericidal activity against both Gram-positive and Gram-negative bacteria [278]. The enzymatic function of PLA_2_ is not the only dependent mechanism for anti-microbial effects [279]. Catalytically inactive Lys49 PLA_2_ homologues, which are non-enzymatic, have also been shown to exert antimicrobial effects by cationic and hydrophobic residues in the C-terminal regions [280].

Beyond its function in envenomation, L-amino acid oxidases from a highly venomous pit viper from South America, *Bothrops mattogrosensis*, or Mato Grosso lancehead, have shown promising anti-microbial activity against Gram-positive strains *S*. *aureus* and *Streptococcus pyogenes* and Gram-negative strains, *E. coli*, *P. aeruginosa*, and *K. pneumoniae*, which are all pathogenic bacteria involved in human diseases, with a growing concern around antimicrobial resistance [281]. LAAO enzyme from king cobra also showed extensive antimicrobial activity against clinical isolates of *E. coli*, *Klebsiella pneumoniae, S. aureus, S. epidermidis*, and *P. aeruginosa* [282]. Generally, LAAO obtained from snakes such as *Trimeresurus jerdonii* and *Trimeresurus mucrosquamatus* has shown promising anti-microbial activity against *B. dysenteriae*, *S. aureus*, *E coli*, and *B. megaterium*, with the possible mechanism associated with hydrogen peroxide release, as the addition of catalase stopped the anti-microbial action [283].

Anti-microbial peptides identified and characterized from snakes have also shown potent activity against clinical and multidrug-resistant isolates [284]. Cardiotoxins are disulfide-rich polypeptides of approximately 60 amino acid residues long, with the ability to damage membranes [285]. CTX 3 is a cytotoxin from the three-finger toxin family from the Taiwan cobra *Naja atra*, and its venom contains multiple isoforms of the cardiotoxin (CTX1-5) [285]. CTX3 is bactericidal against Gram-positive bacteria, *Staphylococcus aureus*, and Gram-negative *Escherichia coli*, with more antibacterial activity against *Staphylococcus aureus* [286]. Toxin γ, which is also a cardiotoxin from *Naja nigricollis*, has bactericidal activity, showing inhibition of *E. coli* and *S. aureus* by inducing bacterial cell membrane permeability [287]. *Naja kaouthia* is another cobra species where cardiotoxins have been experimentally demonstrated to possess antibacterial activity against Gram-positive, *Bacillus subtilis* [288]. Vgf-1 peptide (60 amino acids in length) from *Naja atra*, Chinese cobra, showed inhibition of the multidrug-resistant strain of *Mycobacterium tuberculosis* [282]. Although the mechanism of Vgf-1 peptide is not fully elucidated, the structure consists of three disulphide linkages, which is a common feature of antimicrobial peptides that interact with bacterial cell membranes [289].

Spider venom peptides cause membrane permeabilization, causing antimicrobial activity [266]. Juruin, derived from the yellow-banded pinktoe tarantula, *Avicularia juruensis*, is a 38-residue-long peptide of cationic nature that has promising inhibition of microbial activity against clinical isolates of *Candida* and *Aspergillus niger* [266]. Lycocitin 1 and 2 are antimicrobial peptides isolated from the Chinese wolf spider, *Lycosa singoriensis*, that show significant inhibition against the growth of *S*. *aureus*, *B. subtilis*, *E. coli*, and *P. aeruginosa*, as well as Candida [180,290,291]. There are also linear α-helical and amphipathic peptides isolated from the ant spider, *Lachesana tarabaevi*, which showed antimicrobial activity against *A*. *globiformis*, *B. subtilis*, *E. coli*, and *P. aeruginosa* [266].

## 7. Strategies for Identifying Therapeutic Leads from Animal Venoms

Discovering therapeutic interventions from animal zootoxins requires a systematic and integrative approach that combines advanced omics technologies with functional and translational strategies [4,6]. Techniques such as high-throughput sequencing [292] genomics, structural biology, bioinformatics tools, and transcriptomics enable the identification and characterization of novel bioactive peptides and proteins [10,293]. However, additional steps are required to translate these molecules into viable therapeutic leads.

Combining these methods with platforms such as proteomics [294], lipidomics [295,296], and glycomics [297,298], a technology that has high precision will enable the identification of molecules of potent bioactivity as potential drug targets with clinical relevance [299]. Proteomic analysis is the foundation for the characterization of many venom components, providing information on the structure and sequence of toxins, including post-translational modifications that affect their bioactivity and toxicity [294]. Additionally, genomics and proteomics, when applied with transcriptomics, shed light on the diversity of venom, gland biology, and the evolutionary adaptations that brought about venom toxin diversity [300]. Techniques that can be used for proteomics, such as HPLC and Mass Spectrometry, are essential for sequencing small molecules and peptides [300]. Transcriptomics permits the sequencing of venom gland transcriptomes, which can give more information on gene expression and the different isoforms of a particular toxin, which are otherwise difficult to characterize [301]. Isoform resolution can also be improved using transcriptomics, which can map the differential gene expression of transcripts [302]. Differences between inter- and intra-species venom variation will elucidate predator-prey co-evolution and mechanisms of evolutionary resistance [300,302]. High-throughput screening has enabled the rapid characterization of highly complex biochemical toxin mixtures and has provided information about their pharmacological effects [303]. Nano fractionation of crude venom, in combination with high-throughput screening, validated with coagulation assays, has revealed that quantification and each fraction have coagulant properties [303]. Combining proteomic and transcriptomic profiling with high-throughput screening speeds up the process of identifying toxin classes and gives researchers insight into their functional characteristics, therefore allowing a comprehensive understanding of their biological activity and potential pharmacological effects [303].

Apart from these strategies, the development of eukaryotic cell culture systems has played a fundamental role in toxinology and drug discovery [304,305]. In vitro cultured cell lines provide physiologically relevant platforms for evaluating the cytotoxicity, selectivity, and mechanisms of action of venom-derived compounds [304]. These systems have been particularly valuable in cancer research, where they facilitate the assessment of antiproliferative, pro-apoptotic, and anti-metastatic activities of venom toxins, as well as the identification of molecular targets and signaling pathways involved in their biological effects [306,307]. Furthermore, cell-based assays serve as an essential bridge between in vitro studies and in vivo validation [308]. In silico approaches have also become indispensable tools in venom-based drug discovery by enabling the prediction of toxin–target interactions through molecular docking and molecular dynamics simulations [309,310,311]. These computational techniques provide insights into binding affinity, structural stability, and molecular recognition, thereby facilitating the prioritization and optimization of promising therapeutic leads before experimental validation [312]. In parallel, advances in molecular biology, including gene cloning, recombinant protein expression systems, and protein engineering, have revolutionized the development of venom-derived therapeutics by enabling scalable and standardized production, functional characterization, and structural optimization of bioactive molecules [313,314,315].

Collectively, these multidisciplinary approaches enable a comprehensive understanding of toxin function while facilitating the transition from venom characterization to drug development. By integrating omics technologies, high-throughput screening, cell-based assays, computational modeling, and recombinant expression systems, researchers can systematically identify, validate, and optimize venom-derived molecules as potential therapeutic candidates [299,316].

## 8. Challenges in Translating Venom Bioactive Molecules to Drugs

Although animal venoms have a diverse range of molecules with several pharmacological effects, converting a venom component that is otherwise considered lethal to a therapeutic intervention remains a challenge [6].

### 8.1. Venom Variability and Standardization Challenges

Interspecific and intraspecific venom variability, protein complexity, and heterogeneous venom properties make it difficult to isolate a specific component and characterize it [317]. Several isoforms of a single toxin can exist in venom, with negligible amino acid differences that can completely alter a toxin’s enzymatic activity between species [233,318]. Hence, pinpointing and characterizing these molecular variations remains a considerable challenge. In addition, many venom components are present in minute quantities in crude venom, making the isolation and purification process technically demanding [5,319]. Obtaining sufficient amounts of individual toxins for structural characterization, developing functional assays, evaluating pharmacological relevance, and incorporating them into preclinical studies can therefore be difficult [313,319]. Advances in analytical chemistry, proteomics, and recombinant expression systems have helped overcome some of these constraints; however, purification and characterization of low-abundance venom components remain a bottleneck in venom-based drug discovery [313,319].

Venom composition can also vary among populations of species, due to differences such as geographical location, diet, and epigenetic changes, which can complicate the reproducibility or large-scale sourcing [320,321].

### 8.2. Bioavailability and Pharmacokinetic Limitations

Examples of translational challenges are venom-derived molecules that have shown promising preclinical progress but have failed during further clinical evaluation [147]. One of them is alfimeprase, which showed quick thrombolytic activity but was discontinued in Phase III trials due to safety issues [147]. Similarly, a disintegrin isolated and characterized from *Agkistrodon contortrix* showed anti-tumor and anti-angiogenic effects in preclinical studies, but its clinical translation was limited due to challenges associated with stability, delivery, and production on a large scale [322]. Another example of importance is ancrod isolated from *Calloselasma rhodostoma*, which showed encouraging results for ischemic stroke but failed in the later stages of clinical trials due to inconsistency and high risk of bleeding [323]. These cases highlight a gap between experimental results and successful therapeutic translation [147]. Clinically successful drug candidates such as Ziconotide highlight the limitations of venom-derived drugs, as Ziconotide can only be administered intrathecally [324] due to poor systemic bioavailability and its inability to reach the brain by crossing the blood–brain barrier [325]. Captopril is another example that exhibits relatively promising oral absorption of around 75%; however, it has a low half-life and rapid clearance by the kidneys, which demands frequent dosing [326].

Venom peptides are small disulphide-rich molecules with precise potency and specificity, but there is a possibility of decreased oral bioavailability since there is quick degradation in blood plasma or low ability to reach drug targets, such as those present across the blood–brain barrier [327]. Therefore, recombinant analogues or derivatives with chemical modifications and formulations need to be developed to improve the pharmacokinetic properties; however, this could involve a high cost [328,329].

### 8.3. Toxicity, Safety, and Immunogenicity Concerns

In terms of safety, translation of a venom component into a drug molecule requires overcoming its intrinsic toxic effect, interactions with off-targets, and small therapeutic windows [6]. Potent toxins that have evolved to immobilize prey need careful engineering to reduce the toxicity while retaining their biological activity to keep them as a therapeutic intervention [6]. Additionally, the immunogenic potential that can arise due to the repeated administration of venom-derived proteins is also a cause for concern [330].

### 8.4. Technological, Economic and Regulatory Challenges

Another barrier that stands in the way of translating venom-derived drugs is technological barriers [331]. Although developments in proteomics, transcriptomics, and structural biology tools have enabled toxin discovery, bridging the gap between structural and functional properties, the idea is still challenging due to limited high-throughput screening platforms and incomplete information about many molecular targets in human disease biology [332]. To determine high-resolution structural information for venom-derived molecules, experimental techniques such as X-ray crystallography are required; however, these approaches often demand extensive optimization of purification, protein stability, and crystallization conditions, and many toxins remain difficult to crystallize [333]. Less than 0.01% of venom components that have been completely characterized experimentally, leaving a considerable proportion of potential molecular targets that are unexplored [334]. Structural characterizations rely heavily on homology models and computational predictions, which may not fully capture the unique features of the toxin, holding back predictive accuracy and translational relevance due to the limited availability of experimental data [333]. To add to this, computational predictions are solely based on existing experimental validation, which is currently insufficient [335,336] as characterization of toxins is expensive, technically demanding, and requires complex purification systems with multistep methodical and analytical protocols to fractionate minute molecular mass and low-abundance components, which could potentially be highly bioactive [334]. This lack of experimentally validated structural data can limit mechanistic understanding, make structure-activity relationships ambiguous, and thus affect the rational design of venom-derived therapeutics [333].

Economic and regulatory factors are also a challenge in drug development from venom origins, as it requires significant investment in the synthesis of peptides, recombinant expression systems, and formulation techniques, with the lingering uncertainty of the market size [337]. Manufacturing recombinant toxins derived from venom for therapeutics should have the appropriate post-translational modifications, which remains a significant technical challenge [338,339]. An example of this is the inhibitor cystine knot (ICK) peptides which rely on complex disulphide bond formation, proper protein folding, and structural stability for bioactivity [340]. This points to the necessity of post-translational modification to maintain toxin functionality [341]. Venom components like three-finger toxins may need glycosylation or additional post-translational modifications that cannot be achieved using a bacterial expression system [342,343]. Recombinant proteins produced in prokaryotic hosts may exhibit altered folding, reduced stability, or less biological activity compared to their native counterparts [341]. These limitations require the use of sophisticated eukaryotic expression platforms such as yeast, insect, or mammalian cells, which will increase the cost of production at the risk of low yields, further complicating large-scale manufacturing and therapeutic development [344,345]. This is relevant in snakebite-related molecules, where the global burden of ophidian incidents is higher in low-income regions, reducing commercial incentives. Put together, the challenges show that although venoms are a collective library of bioactive molecules, pharmacological lead molecules that have reached the bedside from the bench are few in number [332,337]. This area of work will be supported actively if drug design, omics technology, and novel drug delivery strategies are integrated. Using this, the urgent global need to address the requirement for new and improved pharmaceuticals for human diseases can be met.

### 8.5. Emerging Solutions: Computational Approaches and Nanotechnology

Integrating advanced computational approaches with experimental work can aid the acceleration of discovery and venom-derived therapeutics [346]. Emerging technologies such as nanotechnology and Artificial intelligence can be used to redefine and revolutionize animal-based drug discovery and development [347]. Involving machine learning, AI-based algorithms, and deep learning, it can connect the analysis of huge data sets of “omics” data to predict toxin structures on an evolutionary basis, as well as predict binding interactions with drug targets in disease biology [348,349,350]. This will aid in the identification of pharmacophores with therapeutic properties and provide structure-activity relationship data, which is essential for lead optimization [351].

Despite the advances in AI-based predictions, many limitations depend on the availability and quality of training datasets [335], which are usually incomplete due to the lack of underexplored species diversity. Optimal drug delivery systems using Nanotechnology, which include nanoparticles, hydrogels, and liposomes, will be ideal to overcome the challenges involved in stability, bioavailability, scalability, and pharmacokinetic properties [41]. Utilization of an effective drug delivery system with nanoparticles or nano-engineered liposomes will increase the specificity towards therapeutic targets and minimize the chances of off-target binding [41]. This will also enable controlled release, drug delivery with a targeted approach, and the crossing of biological barriers like the blood–brain barrier, which are otherwise difficult to reach, which will increase the safety and efficacy [41]. However, challenges include non-covalent interactions between nanoparticles and venom toxin components that could alter their properties as well as become unstable once they enter the body because the nanoparticle-venom biochemistry is highly dependent on pH, ionic interactions, and conjugation could involve alteration to the protein structure, folding, which has a direct impact on bioactivity [334]. Post-translational modifications that are altered or lost in the process of toxin nanoparticle conjugation can also result in the loss or change in their original biological activity [334]. Emerging interdisciplinary frameworks, such as oncovenomics, will integrate venomics and cancer biology to systemically identify and characterize peptides from venom sources that have considerable therapeutic potential [307]. This approach includes proteomics, computational biology, functional assays, and in vitro work to accelerate the translation of therapeutic candidates from venom sources [307].

By converging AI-guided discovery, oncovenomics, and nanobiotechnology, a promising frontier in venom-derived drug discovery is imminent [331,352,353,354,355]. However, this is not without significant challenges related to sufficient quantitative and experimental data, validation, translational feasibility, safety, efficacy, and adherence to regulatory guidelines [147]. The future of venomics research must prioritize integrating AI and computational predictions with high-throughput validation in the laboratory to bridge the much-widened gap between potential drug discovery and translational outcomes.

## 9. Conclusions

Animal venoms are evolutionarily refined arsenals of biologically potent molecules that exert diverse mechanistic actions, including the modulation of ion channels, GPCRs, enzyme inhibition, and the regulation of downstream signalling, resulting in numerous promising leads. Modern venomics, integrating proteomics, transcriptomics, high-throughput screening, and AI-driven pipelines, will enable systematic identification, characterization, and evaluation of their bioactive properties.

However, multifaceted barriers are present, which explain why venom-derived therapies are relatively few, owing to biological and technical hurdles. Safety considerations, regulatory requirements, and the costs associated with engineering venom-derived peptide analogues remain important challenges. Many approaches have been conceptualized to overcome these translational challenges, especially recombinant DNA technology and protein engineering to generate modified toxin variants with reduced toxicity while retaining their desired pharmacological activity. The use of eukaryotic expression systems such as yeast, insect, and mammalian cell cultures has also been discussed, enabling the production of recombinant proteins with the required post-translational modifications. In addition, chemical modifications of the venom-derived therapeutic molecules can be utilized, including cyclization, PEGylation, and amino acid substitutions that could possibly enhance resistance to proteolytic degradation, optimize the pharmacokinetics, and increase bioavailability. Collectively, these strategies can contribute towards the successful translational outcome of venom-derived molecules to clinically viable therapeutics.

Site-directed mutagenesis is another strategy to expand the utility of venom-derived molecules, which aids in the selective modification of amino acid residues that are associated with toxicity while preserving the three-dimensional structure as well as biological function. In this way, engineered recombinant toxins could have diminished toxic effects while maintaining their immunogenicity, making them suitable candidates for producing improved antivenom and for immunization studies. This approach also offers valuable templates for structure-guided drug design and medicinal chemistry, thereby facilitating the development of therapeutic analogues that are safer with improved pharmacological profiles.

Beyond their direct therapeutic applications, toxins from animal venoms can also serve as promising immunomodulatory agents and vaccine adjuvants. Venom-derived molecules could be used to trigger innate and adaptive immune responses, aid antigen presentation, and promote enhanced humoral immunity. These properties make toxin-derived components attractive candidates for vaccination strategies and immunotherapy. Although this area requires considerable exploration and thorough investigation before clinical use, it exemplifies how animal venoms may contribute to future therapeutic innovations. In addition to their roles as therapeutic lead molecules, these toxins are still used as antigens for producing antivenom to treat envenomation. Traditional antivenoms are generated by immunizing equine animals with venoms or venom fractions; however, advances in recombinant DNA technology will enable the production of toxin-derived immunogens, which are standardized, safe, and ethically sustainable alternatives. Recombinant antigens can be a gateway to improving immunization strategies by focusing the immune response on clinically relevant toxins involved in severe systemic envenomation while reducing dependence on venom extraction from animals. These developments will also enable the emergence of next-generation antivenoms and antibody-based therapeutics with higher specificity, safety, and standard manufacturing consistency.

In conclusion, venoms from different animal species represent a vast reservoir of molecular biodiversity and pharmacological potential that remains only partially exploited at the translational level, though significant biological, technical, economic, and regulatory challenges continue to limit their clinical translation. Advances in venomics, recombinant biotechnology, protein engineering, artificial intelligence, and precision drug design are rapidly expanding the possibilities for venom-based therapeutics. Continued integration of these technologies will not only facilitate the discovery of novel drug leads but also support the development of safer therapeutics, improved immunogens, and next-generation antivenoms. Collectively, these advances have the potential to bridge the gap between venom-derived bioactive molecules and clinically effective treatments for a wide range of human diseases.

## Figures and Tables

**Table 1:** Species-specific venom-derived peptides and toxins and their corresponding molecular targets, pharmacological and therapeutic applications. The table is organized to highlight the taxonomic diversity of venom-derived molecules and their potential translational role in drug discovery and development.

Toxin and Source	Toxin Type	Mechanistic Activity	Potential Pharmacological Applications	Ref.
**Snakes**				
Cardiotoxins CTX1,2,3,4,5 (*Naja oxiana*, *Naja naja atra*)	Three-finger toxin (3FTx), cytotoxin	Pore formation in cell membranes.	Anticancer, antimicrobial (cytolytic agents).	[161]
α-Bungarotoxin (*Bungarus multicinctus*)	Three-finger toxin (3FTx), α-neurotoxin	Targets muscular (α1) and neuronal (α7) nAChRs, blocks neuromuscular transmission	nAChR probe for neuromuscular and receptor-binding studies	[162]
Haditoxin (*Ophiophagus hannah*)	Three-finger toxin (3FTx), α-neurotoxin	Preferentially blocks muscle (α1) and neuronal nAChRs (α7, α3β2, α4β2), inhibits cholinergic neurotransmission.	Research tool/lead molecule for probing nicotinic receptor subtypes (especially α7) and for inspiring α7-targeted drug design.	[163]
κ-neurotoxins (*Bungarus multicinctus*)	Three-finger toxin (3FTx), κ-neurotoxin	Blocks neuronal nAChRs (especially α3β2 subtype) and modulates synaptic transmission.	Selective modulators for nAChR-targeted neuropharmacology	[164,165,166]
Oh 9-1 (*Ophiophagus hannah*)	Ω-neurotoxin	nAChr antagonist Blocks the binding of acetylcholine and inhibits synaptic transmission	Probe to study nAChr, lead for receptor modulation	[167]
Mambalgins (*Dendroaspis polylepis*)	Three-finger toxin (3FTx) family	Block Acid-Sensing Ion Channels (ASIC).	Non-opioid analgesic targeting the ASIC channels.	[168,169,170]
Calciseptine (*Dendroaspis polylepis*)	Three-finger toxin (3FTx) family	Blocks L-type Ca^2+^ channels, inhibiting calcium influx and smooth muscle contraction.	Template for calcium channel blockers in cardiovascular therapy.	[171]
Calliotoxin (*Calliophis bivirgatus*)	Kunitz-type peptide	Activates voltage-gated sodium channels (NaV1.7) inhibits inactivation, and causes sustained neuronal excitation.	Tool for studying NaV1.7-mediated pain pathways.	[172,173]
Textilinin-1 and 2 (*Pseudonaja textilis*)	Kunitz-type serine protease inhibitor	Reversible, tight-binding & inhibition of Plasmin.	Bleeding disorders (antifibrinolytic)	[174,175]
Lebetin (*Macrovipera lebetina*)	Venom natriuretic peptide (NP family)	Activates natriuretic peptide receptors, increasing cGMP and inducing vasodilation.	Cardioprotective peptide for myocardial ischemia and heart failure.	[176,177]
Dendroaspin *(Dendroaspis jamsonii*)	disintegrin-like protein	Targets integrins αIIbβ3, αVβ3 and α5β1 on platelets via an RGD motif.	Antithrombotic therapy and cancer metastasis inhibition.	[178,179]
**Spiders and Anemones**				
µ-TRTX-HI1a (*Haplopelma lividum*)	Inhibitor Cystine Knot (ICK)	inhibits voltage-gated sodium channel (NaV1.8), reduces sodium current amplitude, decreases excitability of pain-sensing neurons.	Selective, non-opioid analgesic for inflammatory and neuropathic pain.	[180]
GsMTx4 *(Grammostola spatulate*)	ICK	Inhibits Piezo (mechanosensitive ion channels).	Potential applications in arrhythmias, muscular dystrophy, pain, and mechanotransduction research.	[180]
µ-TRTX-Hhn2b *(Selenocosmia hainana*)	ICK	weak inhibition of voltage-gated sodium channels (NaV1.7), reduces sodium current amplitude without altering channel gating.	Lead scaffold for development of NaV1.7-targeting analgesics	[173]
Huwentoxin-IV *(Ornithoctonus huwena*)	ICK	Inhibits NaV1.7	Promising lead for non-opioid analgesic drug development.	[181]
Dalazatide (*S. helianthus*)	ShK analogue	Blocks Voltage-gated potassium channel Kv1.3, suppresses effector memory T cell activation.	Promising lead for autoimmune diseases.	[182]
**Leeches**				
Poecistasin (*Poecilobdella manillensis*)	Kunitz-type serine protease inhibitor	targets factor XIIa and kallikrein	Antifibrinolytic agent and as a scaffold for protease targeting drugs.	[183,184]
Antistasin (*Haementeria* *Officinalis*)	Antistasin family (Serine protease inhibitors)	Inhibits Factor Xa, blocks thrombin generation.	Prototype for anticoagulant drug development.	[185]
Hirustasin and Bdellastatin (*Hirudo medicinalis*)	Antistasin family & Kazal-type serine protease inhibitor, respectively.	hirustasin targets kallikrein, Bdellastatin inhibits trypsin-like proteases.	anti-inflammatory applications in inflammation, hemostasis research, and reproductive biology.	[186,187]
**Scorpion**				
Charybdotoxin (ChTX) *(Leiurus quinquestriatus*)	Ion channel modulator	blocking the potassium channels	Cardiovascular and neurological disorders	[188]
Iberiotoxin (IbTX) *(Buthus tamulus*)	Ion channel modulator	blocking large conductance calcium- activated potassium channels	Cardiovascular and neurological disorders	[189]
Margatoxin (MgTx) *Centruroides margaritatus*	Ion channel modulator	Kv1.3 channel blockers	T cell regulation, autoimmune disorders	[190]
Vm24 (*Vaejovis mexicanus smithi*)	Ion channel modulator	Kv1.3 channel blockers	T cell regulation, autoimmune disorders	[191,192]
Chlorotoxin *(Leiurus quinquestriatus*)	Specific to cancer cells	Glioma	Anti-cancer activity	[193]
**Snails**				
GIIIA (*Conus geographus*)	Ion channel modulator	block voltage-gated sodium channels	Prevent neuropathic and inflammatory pain	[194]
PIIIA (*Conus purpurascens*)	Ion channel modulator	block voltage-gated sodium channels	Prevent neuropathic and inflammatory pain	[194]
PVIA (*Conus purpurascens*)	Ion channel modulator	targets sodium channels	Promising treatment for epilepsy	[195]
PVIIA *(Conus purpurascens*)	Ion channel modulator	Acts on voltage-gated calcium channels	Neuronal excitation	[196]

## Data Availability

No new data were created or analyzed in this study.

## Abbreviations

The following abbreviations are used in this manuscript:

ACE	Angiotensin-Converting Enzyme
AI	Artificial Intelligence
ANP	Atrial Natriuretic Peptide
AT1AR	Angiotensin Type-1A Receptor
AT1R	Angiotensin Type-1 Receptor
AT2R	Angiotensin Type-2 Receptor
ATIB	Angiotensin Type-1B Receptor
BBB	Blood–Brain Barrier
BPF	Bradykinin-Potentiating Factors
CaTxs	Cardiotoxins
CGRP	Calcitonin Gene-Related Peptide
CTX	Cytotoxin
DVT	Deep Vein Thrombosis
FAD	Flavin Adenine Dinucleotide
FMN	Flavin Mononucleotide
GIP	Glucose-Dependent Insulinotropic Polypeptide
GLP-1	Glucagon-Like Peptide-1
HPLC	High-Performance Liquid Chromatography
IKβ	IκB Kinase
Kv	Voltage-Gated Potassium Channel
LAAO	L-Amino Acid Oxidase
mAChRs	Muscarinic Acetylcholine Receptors
nAChRs	Nicotinic Acetylcholine Receptors
NaV	Voltage-Gated Sodium Channel
CaV	Voltage-Gated Calcium Channel
NP	Natriuretic Peptides
RAAS	Renin–Angiotensin–Aldosterone System
RGD	Arg-Gly-Asp Motif
ROS	Reactive Oxygen Species
SRTXs	Sarafotoxins
3FTxs	Three-Finger Toxins
NaV1.7	Voltage-Gated Sodium Channel 1.7
P/Q	P/Q-Type Calcium Channel
N-type	N-Type Calcium Channel
R-type	R-Type Calcium Channel
L-type	L-Type Calcium Channel
M1–M5	Muscarinic Receptor Subtypes 1–5
ShK	Stichodactyla helianthus Potassium Channel Toxin
HWTX-IV	Huwentoxin-IV
HNTX-I	Hainantoxin-I
PhTx3	Phoneutria nigriventer toxin-3
Oh9-1	Omega Neurotoxin-9-1
GsMTx4	Grammostola spatulata Mechanotoxin-4
HL-60	Human Promyelocytic Leukemia Cells
MCF-7	Michigan Cancer Foundation-7 Breast Cancer Cells
DU-145	Prostate Cancer Cell Line
HepG2	Human Hepatocellular Carcinoma Cells
A549	Human Lung Carcinoma Cells
T24	Human Bladder Carcinoma Cells
BIU-87	Human Bladder Cancer Cells
Calu-6	Human Lung Adenocarcinoma Cells
Molt-4	Human T-Lymphoblastic Leukemia Cells
C8166	Human T-Cell Line
